# Dopamine Genetic Risk Score Predicts Depressive Symptoms in Healthy Adults and Adults with Depression

**DOI:** 10.1371/journal.pone.0093772

**Published:** 2014-05-16

**Authors:** Kristin M. Pearson-Fuhrhop, Erin C. Dunn, Sarah Mortero, William J. Devan, Guido J. Falcone, Phil Lee, Avram J. Holmes, Marisa O. Hollinshead, Joshua L. Roffman, Jordan W. Smoller, Jonathan Rosand, Steven C. Cramer

**Affiliations:** 1 Department of Anatomy and Neurobiology, University of California Irvine, Irvine, California, United States of America; 2 Center for Human Genetic Research, Massachusetts General Hospital, Boston, Massachusetts, United States of America; 3 Department of Psychiatry, Massachusetts General Hospital/Harvard Medical School, Boston, Massachusetts, United States of America; 4 Stanley Center for Psychiatric Research, The Broad Institute of Harvard and MIT, Cambridge, Massachusetts, United States of America; 5 Department of Psychology, Yale University, New Haven, Connecticut, United States of America; 6 Department of Psychology, Center for Brain Science, Harvard University, Cambridge, Massachusetts, United States of America; 7 Department of Neurology, Massachusetts General Hospital/Harvard Medical School, Boston, Massachusetts, United States of America; 8 Program in Medical and Population Genetics, The Broad Institute of Harvard and MIT, Cambridge, Massachusetts, United States of America; 9 Department of Neurology, University of California Irvine, Irvine, California, United States of America; Kunming Institute of Zoology, Chinese Academy of Sciences, China

## Abstract

**Background:**

Depression is a common source of human disability for which etiologic insights remain limited. Although abnormalities of monoamine neurotransmission, including dopamine, are theorized to contribute to the pathophysiology of depression, evidence linking dopamine-related genes to depression has been mixed. The current study sought to address this knowledge-gap by examining whether the combined effect of dopamine polymorphisms was associated with depressive symptomatology in both healthy individuals and individuals with depression.

**Methods:**

Data were drawn from three independent samples: (1) a discovery sample of healthy adult participants (n = 273); (2) a replication sample of adults with depression (n = 1,267); and (3) a replication sample of healthy adult participants (n = 382). A genetic risk score was created by combining functional polymorphisms from five genes involved in synaptic dopamine availability (*COMT* and *DAT*) and dopamine receptor binding (*DRD1*, *DRD2*, *DRD3*).

**Results:**

In the discovery sample, the genetic risk score was associated with depressive symptomatology (β = −0.80, p = 0.003), with lower dopamine genetic risk scores (indicating lower dopaminergic neurotransmission) predicting higher levels of depression. This result was replicated with a similar genetic risk score based on imputed genetic data from adults with depression (β = −0.51, p = 0.04). Results were of similar magnitude and in the expected direction in a cohort of healthy adult participants (β = −0.86, p = 0.15).

**Conclusions:**

Sequence variation in multiple genes regulating dopamine neurotransmission may influence depressive symptoms, in a manner that appears to be additive. Further studies are required to confirm the role of genetic variation in dopamine metabolism and depression.

## Introduction

Although depression is one of the most prevalent and costly psychiatric conditions, estimated to affect 16.6% of US adults [1], knowledge of its etiology remains limited. Currently, the most commonly articulated theory regarding the pathophysiology of depression focuses on systems regulating monoamine neurotransmission [2]. This theory postulates that dysregulation of monoamine neurotransmission increases susceptibility to depression. Serotonin has been the most frequently studied monoamine to date. However, evidence from human and animal studies suggests that other monoamines, particularly dopamine, may be involved [3], [4], [5], [6], [7], [8], [9], [10], [11]. Chronic antidepressant treatment potentiates the dopamine system [3]. In rodents, dopaminergic neurons modulate depressive symptoms [4]. Disturbances in limbic dopaminergic pathways may contribute to depressive symptoms in Parkinson’s disease (PD) [5], and dopaminergic therapy for PD can reduce depressive symptoms even in the absence of formal antidepressant treatment [6]. Based on such findings, dopaminergic targets have become a focus for depression therapies; one study found that the DRD2 agonist pramipexole was as effective as fluoxetine in the treatment of MDD [12]. Thus, a decreased level of endogenous dopaminergic neurotransmission might make a significant contribution to depression pathology. Another method by which decreased dopaminergic neurotransmission might increase depressive symptoms is through its influence on motivation and reward processing, both of which are impaired in depression [13], [14] and are strongly linked to the dopamine system [15], [16], [17], [18].

Examination of the effect of dopamine-related genetic variants may extend knowledge of the role of dopamine neurotransmission in the etiology and course of depression. This line of research is warranted, as depression is highly heritable [19] and several genetic variants have been found to modulate endogenous dopamine neurotransmission [20], [21], [22], [23]. Thus far, evidence on the role of variation in dopamine neurotransmission in depression has been mixed. While some studies find that dopamine-related variants are associated with multiple psychiatric and neurological diseases [24],[25],[26],[27],[28],[29], other studies find no association [9], [30]. Moreover, when dopamine-related polymorphisms have been studied in the context of genome-wide association studies (GWAS), none have emerged as significantly associated with depression [31], [32], [33]. One likely contributor to these inconsistent findings is that common genetic variants for complex disease tend to have small to modest effects. Thus, tests of association based on a single nucleotide polymorphism (SNP) are unlikely to yield significant effects unless very large samples are studied.

We sought to provide additional evidence regarding the role of dopamine in depression by examining the combined effect of five dopamine-related polymorphisms and depressive symptom severity. We used a genetic risk score approach, which sums the effects of multiple polymorphisms in the same biological system. Genetic risk score approaches have been informative in several medical [34], [35] and psychiatric [36], [37] settings, including when studying the role of dopamine [38], [39], [40]. The genetic risk score employed in the current study captures genetic variation in several aspects of the brain dopamine system, including synaptic dopamine availability (*COMT* and *DAT*) and dopamine receptor binding (*DRD1*, *DRD2*, *DRD3*). These proteins are abundant in the cortical and subcortical neural structures affected in depressive disorders [10], [11]. The genetic risk score employed in this analysis has been linked to learning a motor skill and the extent to which oral L-dopa supplementation improves this learning [39].

## Materials and Methods

### Overview

The main hypothesis we sought to test was that genetic variation in the dopamine system was significantly related to depressive symptoms, with genotypes corresponding to lower dopamine neurotransmission being associated with greater depressive symptomatology. We further hypothesized that the genetic risk score would have a stronger relationship to depression than would any single polymorphism, as the effects of multiple polymorphisms acting on the same neural system are hypothesized to be additive. We tested these hypotheses in a discovery sample of prospectively enrolled healthy participants and in two replication samples. The first replication cohort comprised individuals diagnosed with major depressive disorder (MDD) from the Sequenced Treatment Alternatives to Relieve Depression Study (STAR*D) [41], [42], [43]. The second replication was attempted using healthy subject data from the Brain Genomics Superstruct Project (GSP) [44], [45].

### Discovery Sample: Healthy Young Adults

The discovery study was conducted in healthy young adults between the ages of 18–35. This Healthy Study (HS) included 273 participants who were recruited from the University of California, Irvine campus and surrounding areas.

#### Protocol

Participants were eligible for the HS if they were between ages 18–35, right-handed, not taking dopamine-activating medications, and free of any current or past major neurological or psychological disorder, assessed by self-report. Participants underwent a blood draw for DNA collection and completed a battery of supervised self-reported measures on health history, demographic data, and the 20-item Center for Epidemiologic Studies Depression Scale (CES-D). The CES-D is a widely used, reliable, and valid measure of depression designed for use in population-based studies [46]. Higher CES-D scores indicate higher levels of depressive symptoms in the past week.

#### Ethics statement

Participants provided written informed consent. The HS was approved by the Institutional Review Board of the University of California, Irvine.

#### Genotyping

DNA was extracted from whole blood by salt precipitation. Genotyping for all polymorphisms was performed using polymerase chain reaction (PCR) - restricted fragment length polymorphism analysis. PCR products were digested with the appropriate restriction enzymes, digestion products were run on agarose gel and then visualized with ethidium bromide. Choice of primer sequences and digestion enzymes followed established protocols for the five polymorphisms examined: *COMT* rs4680 [47], *DAT* rs28363170 [48], *DRD1* rs4532 [49], *DRD2/ANKK1* rs1800497 [50], and *DRD3* rs6280 [51].

#### Calculating the dopamine genetic risk score

A dopamine genetic risk score was created, representing the additive effect of five polymorphisms related to dopaminergic neurotransmission. A total of five polymorphisms were selected from literature review based on (1) minor allele frequency >0.25 and (2) strong association with biological measures ([39], see also below). Participants were given a score of one for each allele present that increases dopamine neurotransmission (Table 1). Genetic risk scores could thus range from zero (lowest basal dopamine neurotransmission) to 10 (highest basal dopamine neurotransmission).

**Table 1:** Summary of polymorphisms and classification for the genetic risk score.

Scoring System	DRD1	DRD2	DRD3	COMT	DAT
	(rs4532)	(rs1800497)	(rs6280)	(rs4680)	(rs28363170)
**0**	A/A	Lys/Lys	Ser/Ser	Val/Val	10/10
**1**	A/G	Glu/Lys	Ser/Gly	Val/Met	9/10
**2**	G/G	Glu/Glu	Gly/Gly	Met/Met	9/9

To create the genetic risk score, participants had one point added to their score for each dopamine-increasing allele that they possess at each of 5 polymorphic sites. This method is very similar to that used by Stice et al [40], Nikolova et al [38] and Pearson-Fuhrhop et al [39].

#### Classification of dopamine variants

1. *COMT* (rs4680, chromosome 22): *COMT* is an enzyme that degrades catecholamines such as dopamine, and has a val^158^met polymorphism in which the val allele results in a protein with 3–4 times lower enzymatic activity, and thus higher dopaminergic tone [22]. In a positron emission tomography (PET) study, F-Dopa metabolism was greater in individuals with the *COMT* val/val genotype, compared to met/met, within several cortical areas [52]. This suggests that dopamine is metabolized faster, and therefore less available, in individuals with the val/val genotype. Presence of the ^158^met variant has been associated with greater working memory and more efficient prefrontal cortex physiology in humans [24]. Each ^158^met allele increases dopamine neurotransmission. Therefore, one point was added to a participant’s genetic risk score for each Met allele present (e.g., A/A genotype was coded as 2; A/G genotype was coded as 1) and Val/Val individuals (G/G genotype) were given a score of 0. In the HS sample, a total of 38 participants had the Met/Met genotype, 129 Val/Met and 106 had Val/Val.

2. *DAT* (rs28363170, chromosome 5): *DAT* is an enzyme that removes synaptic dopamine. The gene that encodes *DAT* has a 40 bp variable number of tandem repeats (VNTR) at the 3′ untranslated region that commonly occurs in either 9 or 10 repeats. Several studies have shown that the 10-repeat allele is related to higher expression of the *DAT* gene, which results in lower dopaminergic tone [20]. Increased *DAT* activity is implicated in the pathology of attention deficit hyperactivity disorder (ADHD), considered a hypodopaminergic state, and stimulants such as methylphenidate, which inhibit *DAT* and increase dopamine levels, are often effective in treating ADHD. Consistent with these findings, the 10-repeat allele has been associated with ADHD [53]. Given these findings, participants received a score of one for each 9 repeat, which produces a relatively higher level of dopamine neurotransmission; those with the 10/10 genotype were therefore scored as zero. In the HS sample, 97.1% had either 10/10, 9/10, or 9/9 genotypes. However, 2.9% of participants (n = 8) had larger (11) and smaller (6, 7, 8) repeats. DAT 11-repeat alleles behave more similarly to 10-repeat alleles [54], and another study found that both 7- and 9-repeat alleles resulted in less DAT activity than the 10-repeat allele [55]. Therefore, larger repeats were coded as 0 and smaller repeats were coded as 1. In the HS sample, a total of 190 participants had the 10/10 genotype, 66 had 9/10, 8 had 9/9, 2 had 11/10, 4 had 6/10, 1 had 7/10 and 1 had 8/10.

3. *DRD1* (rs4532, chromosome 5): *DRD1* is a dopamine receptor and its gene has a −48 A/G SNP in the 5′ untranslated region [56]. Some studies suggest that the *DRD1* G allele may be associated with increases in brain dopamine neurotransmission. For example, the G allele is more common in persons with bipolar disorder [28], is associated with an increased rate of nicotine dependence [25], and has been implicated in traits such as compulsive eating, shopping, and gambling, all of which are linked to increased brain dopaminergic tone [26]. For these reasons, participants had one point added to their score for each G allele (e.g., G/G genotype received a score of 2; A/G genotype received a score of 1; A/A genotype received a score of 0). In the HS sample, a total of 21 participants had the G/G genotype, 101 had G/A and 151 had A/A.

4. *DRD2* (rs1800497, chromosome 11): The *ANKK1* TaqIA polymorphism, a Glu to Lys substitution at position 713 of the *ANKK1* protein, near *DRD2*, is associated with a 30–40% reduction in striatal D2 binding in post-mortem brain tissue, with Lys carriers showing significantly lower D2 binding than Glu/Glu homozygotes [23]. PET st udies have also found reduced striatal D2 receptor availability with the Lys allele [57]. The Lys allele is also associated with predisposition to neuroleptic malignant syndrome, a hypodopaminergic state [27]. This suggests that behaviorally, the Lys allele is more common in individuals who have a condition characterized by an overall decrease in brain dopaminergic signaling. Despite the opposing molecular effects of dopamine binding at D1 and D2 receptors, behavioral evidence suggests that DRD1 and DRD2 act in synergy [58], [59], [60], [61], and that the regulatory balance of dopamine signaling is optimized when these two receptor types work in concert [58]. Therefore, a polymorphism that decreases the amount of D2 receptor availability and/or binding will likely still have the same behavioral effect as one that decreases central dopaminergic activity, as seen with the association between the A1 allele and neuroleptic malignant syndrome [27]. Given these findings, one point was added to a participant’s score for each Glu (A2) allele present (e.g., G/G genotype received a score of 2; A/G genotype received a score of 1; A/A genotype received a score of 0). In the HS sample, a total of 23 participants had the Lys/Lys genotype, 115 had Lys/Glu and 135 had Glu/Glu.

5. *DRD3* (rs6280, chromosome 3): *DRD3* is a dopamine receptor that has a SNP resulting in a Ser to Gly substitution at position 9 of the protein. Dopamine has an affinity to the Gly variant that is 4–5 times higher than its affinity to the Ser variant, and in response to dopamine the Gly variant more robustly increases cAMP inhibition [21]. Initial classification of the ^9^Gly *DRD3* polymorphism was based in part on a study that reported an increased risk of tardive dyskinesia, a dopamine supersensitive state [62], although this association was less clear upon subsequent meta-analysis [63]. Further support for this classification of the ^9^Gly *DRD3* allele comes from Savitz et al, who found that participants with the ^9^Gly allele showed increased striatal reward-related dopamine release during a gambling task [64]. Any presence of the ^9^Gly *DRD3* variant increases dopamine neurotransmission, and thus one point was added for each Gly allele present (e.g., C/C genotype received a score of 2; C/T genotype received a score of 1; T/T genotype received a score of 0). In the HS sample, a total of 32 participants had the Gly/Gly genotype, 123 had Gly/Ser and 118 had Ser/Ser.

#### Data analysis

Statistical tests were performed using JMP 8 statistical software (SAS, Cary, NC). Linear regression analysis was used to examine the association between the genetic risk score and depressive symptoms, adjusting for self-reported race/ethnicity. A second linear regression model was used to assess the effect of the genetic risk score on depression, controlling for age (continuous) and gender (0 = male, 1 = female) as well as race/ethnicity. Next, a “leave one out” approach was examined to determine if the association between the genetic risk score and depression remained significant when removing each gene from the score, one at a time; this approach tests whether any single variant is driving the associations with the dopamine genetic risk score. In all cases, race/ethnicity was included as a covariate. Hardy-Weinberg equilibrium was confirmed for each gene using Chi-squared tests. All participants had full genetic data available. CES-D scores were slightly skewed towards lower values; as skew and kurtosis values were within reasonable limits (skew = 1.5, kurtosis = 3.3 [65]), we did not conduct any transformations of the data.

### Replication Sample: STAR*D

The first replication attempt used data from the Sequenced Treatment Alternatives to Relieve Depression Study (STAR*D; [41], [66]), which was designed to determine the psychopharmacological treatments most effective in treating depression.

#### Protocol

STAR*D was conducted at 41 clinical sites, representing primary care or psychiatric outpatient clinics, across the United States over a period of 37 months. The study only enrolled individuals seeking treatment. To be eligible, participants were required to have a clinical diagnosis of non-psychotic major depressive disorder confirmed with a DSM-IV checklist and also have a current score of 14 or higher on the 17-item Hamilton Rating Scale of Depression (HAM-D; [67]). Higher HAM-D scores indicate higher levels of depressive symptoms. To maximize generalizability of the study results, participants with most comorbid psychiatric and medical conditions were not excluded. However, participants were excluded if they had a lifetime diagnosis of major depressive disorder with psychotic features, schizophrenia, schizoaffective disorder, or bipolar disorder I, II, or not otherwise specified based on clinical assessment and self-report (but not self-report questionnaires). A total of 4,041 participants aged 18–75 were enrolled in STAR*D; 1,953 consented to provide DNA samples for genomic analyses (see for example [68], [69]). To control for the effect of race/ethnicity, the current replication analysis was restricted to the 1,267 individuals who self-reported being White. Data for depressive symptoms was taken from baseline, prior to treatment.

#### Ethics statement

Participants provided written, informed consent. The STAR*D study was approved and monitored by the institutional review boards at each participating institution involved in the study, a National Coordinating Center, a Data Coordinating Center, and the Data Safety and Monitoring Board at the National Institute of Mental Health.

#### Genotyping

Genotyping was conducted on two different platforms. About half of the sample was genotyped with the Affymetrix GeneChip Human Mapping 500 K Array; the second half was genotyped using the Affymetrix Genome-Wide Human SNP 5.0 Array. A total of 500,568 SNPs were assayed by both arrays. None of the genetic variants of interest in this study were directly genotyped in STAR*D, though three SNPs (rs4680, rs1800497, rs6280) were imputed from the HapMap (CEU reference panel, release 23) using BEAGLE 3.3 [66]. These three SNPs had imputation quality score of R^2^ of >0.80.

#### Calculating the dopamine genetic risk score

Dosage scores, obtained from imputation, were used to calculate the genetic risk score, which was the sum of the risk allele counts for the three variants included in this study. Higher values correspond to greater levels of brain dopamine neurotransmisson. Only participants with complete data for all variants were included in the genetic risk score. A total of 126 respondents, or 9.94% of the sample, were eliminated based on this criterion.

#### Data analysis

Genetic risk scores were calculated using PLINK version 1.07. Analyses were conducted with SAS version 9.2, using a two-tailed alpha = 0.05 for significance. Univariate analyses were performed to describe the distribution of the genetic risk scores, depressive symptoms, and demographic characteristics. A linear regression model was then fit to estimate the association between the genetic risk score and total symptoms of depression. Depressive symptom scores were normally distributed. All analyses controlled for age (continuous), sex (0 = male; 1 = female), marital status (0 = married/cohabiting; 1 = never married; 2 = divorced, widowed, or separated), and principal components for genetic ancestry. Data were cleaned using strict quality control methods as described elsewhere [44].

### Second Replication Sample: GSP

The second replication effort used data from the Brain Genomics Superstruct Project (GSP), a large-scale study focusing on the links between genes, brain function, and behavior in healthy young adults [44], [45].

#### Protocol

The GSP recruited healthy, native English-speaking young adults, ages 18–35, from sites across Boston and surrounding communities. To be eligible to participate, participants must not have had a history of head trauma, current or past Axis I pathology or neurological disorder, current or past use of psychotropic medications, acute physical illness, or a head injury with loss of consciousness. Eligible participants completed a structural and functional magnetic resonance imaging (MRI) scan, provided a saliva sample (Oragene DNA Genotek) for the purpose of DNA collection, and completed a set of health and demographic questionnaires on the day of their MRI appointment. Participants were also invited to complete a set of optional web-based instruments that measured personality, intelligence, mood, and behavior. Mood was assessed using five items from the shortened-version of the Profile of Mood States (POMS), which in its complete form is a 30-item scale designed to assess affective mood states, including depression, tension, anxiety, anger, hostility, and confusion [70]. Respondents described their mood in the past week using a Likert-scale (0 = not at all to 4 = extremely). Numerous factor analytic studies have derived six-subscales or factors corresponding to these 30 items on the POMS (see for example [70], [71]; these six subscales are: tension-anxiety; depression-dejection; anger-hostility; fatigue-inertia; vigor-activity; confusion-bewilderment. We examined the normalized scores (t-scores) of the depression/dejection subscale of the POMS, which consisted of five items and had good internal consistency reliability (>0.90) [70], [72].

#### Ethics statement

Investigators obtained written informed consent from participants. All study procedures were approved by the Institutional Review Board at Partners Health Care and Harvard University.

#### Genotyping

Participants were genotyped using the Illumina Infinium OMNI 1 quad chip, which after quality control captured 763,104 SNPs. Three of the genetic variants of interest in the current study were directly genotyped (rs4680, rs6280, and rs4532); one was imputed (rs1800497). A total of 470 participants were genotyped, all of whom were White by self-report; of these, 442 had genetic data that passed quality control. Imputation was completed using MACH (http://www.sph.umich.edu/csg/abecasis/MACH/tour/imputation.html) and the 1000 Genomes European data (phase 1 release v3) was used as the reference. The ChunkChromosome program was used to split each chromosome into 10 pieces, for which all GSP participants were imputed as one batch. The imputation quality score R^2^ of 0.80 was used in filtering; all imputed SNPs had R^2^>0.99.

#### Calculating the dopamine genetic risk score

The genetic risk score was calculated from four of the available variants using PLINK version 1.07. Only participants with complete genetic, phenotypic, and covariate data were included. Based on these criteria, 381 respondents were included in the analysis (13.8% were excluded).

#### Data analysis

We conducted analyses with SAS version 9.2 (alpha 0.05 was the level of statistical significance). We began by conducting univariate analyses to describe the distribution of the scores, depressed mood, and demographic characteristics. We then fit a linear regression model to estimate the association between the genetic risk score and depressed mood score. Depressed mood scores were slightly skewed towards lower values; as skew and kurtosis values were within reasonable limits (skew = 1.16; kurtosis = 1.01) [65], we did not conduct any transformations. All analyses controlled for age (continuous), sex (0 = male; 1 = female), and principal components for genetic ancestry. As described previously [68], the data were cleaned using several quality control standards.

## Results

### Discovery Cohort: Healthy Young Adults

**Figure 1:**
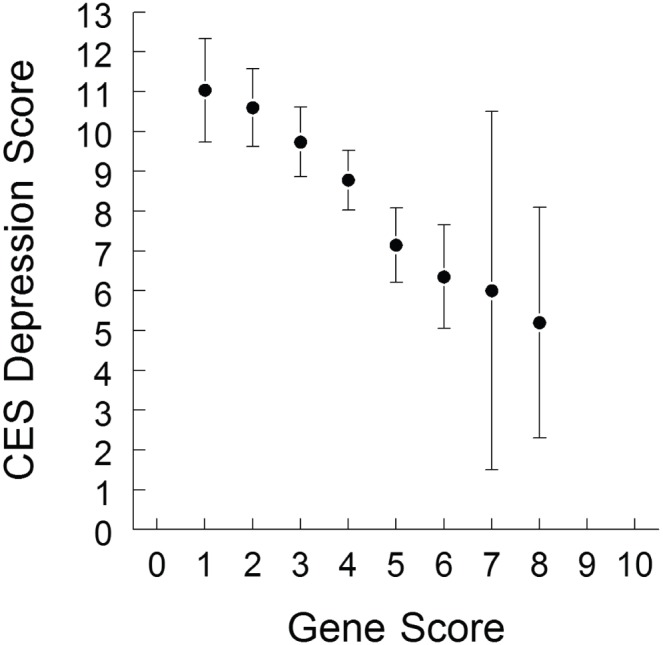
CES-D score by genetic risk score for the population of HS participants. Results are mean ± SE.

Demographic characteristics of participants in the HS sample are presented in Table 2. The average CES-D score (out of a possible range of 0–60) was 8.7±6.5. The sample was predominately Asian (49%) and White (30%). All polymorphisms were in Hardy-Weinberg equilibrium (rs4680: χ^2^ = 0.016, p = 0.90; rs4532: χ^2^ = 0.51, p = 0.48; rs6280: χ^2^ = 0.00, p = 0.995; rs1800497: χ^2^ = 0.046, p = 0.83). In the HS sample, the dopamine genetic risk score was significantly associated with CES-D score after adjusting for race/ethnicity (β = −0.80, p = 0.003), with lower genetic risk scores corresponding to greater levels of depression (Figure 1, Table 3). When controlling for the additional covariates sex and age, the association between genetic risk score and CES-D score remained significant (β = −0.75, p = 0.005). When the 8 participants with uncommon DAT genotypes were excluded from analyses, these associations remained significant (β = −0.79, p = 0.004; β = −0.74, p = 0.006).

**Table 2:** Demographic and baseline data.

	HS	STAR*D	GSP
**N**	273	1267	381
**Age**	20.8±2.8	43.5±13.5	21.37±3.13
**% Female**	56.8% (N = 155)	58.5% (N = 745)	58% (N = 221)
**Average Genetic risk score**	3.7±1.6 (1–8)	1.63±0.6 (0–3)	1.48±0.64 (0, 3.5)
**Polymorphisms included**	rs4680	rs4680*	rs4680
	rs4532	rs6280*	rs4532
	rs6280	rs1800497*	rs6280
	rs1800497		rs1800497*
	rs28363170		

Data are shown as mean ± SD (range).

*Denotes the SNP was imputed.

**Table 3:** Adjusted association between dopamine score and depressive symptoms.

	Beta	s.e.	p-value	95% CI
**HS**	−0.80	0.27	0.003	−1.3, −0.27
STAR*D	−0.51	0.25	0.04	−0.99, −0.01
**GSP**	−0.86	0.60	0.15	−2.03, 0.31

Cell entries are beta coefficients, standard errors (s.e.), p-values and 95% confidence intervals (CI). The HS model controlled for race/ethnicity. The STAR*D model contained controls for age (continuous), sex (0 = male; 1 = female); marital status (0 = married/cohabiting; 1 = never married; 2 = divorced, widowed, or separated); and five principle components for genetic ancestry/population stratification. The GSP model controlled for age (continuous), sex (0 = male; 1 = female), and four principle components for genetic ancestry/population stratification. Depressive symptoms were measured by 3 scales: CES-D (HS), HAM-D (STAR*D), POMS short form (GSP).

We conducted several exploratory analyses in the HS population, including an examination of single variant effects, as well as a set of 4-gene genetic risk scores generated using a “leave one out” approach. All of these exploratory models controlled for race/ethnicity. In the examination of single variant effects (see Table 4), we found that two individual variants were correlated with CES-D score: the *DRD2/ANKK1* Lys (A1) allele (β = −1.6, p = 0.01), and the *DRD3* Ser allele (β = −1.1 p = 0.06) were each individually associated with higher depression scores, though only *DRD2/ANKK1* was statistically significant and remained so after adjusting for multiple comparisons using a Bonferroni-corrected α = 0.01. Thus the genetic risk score showed a greater statistically significant relationship to depression than was found with any single polymorphism. Finally, results of the “leave one out” approach, in which we removed one variant at a time from the genetic risk score, revealed that the adjusted association between the genetic risk score and depression remained significant for all five of the 4-gene iterations of the dopamine genetic risk score. Thus no single variant appeared to be driving the score, as the genetic risk score remained statistically significant when omitting any one gene, including *DRD2/ANKK1*.

**Table 4:** Adjusted association between individual dopamine variants and depressive symptoms.

	HS Sample			
	Beta	s.e.	p-value	95% CI
rs4680	−0.39	0.59	0.51	−1.55, 0.77
rs4532	−0.26	0.64	0.69	−1.62, 1.10
rs6280	−1.1	0.59	0.06	−2.26, 0.06
rs1800497	−1.6	0.62	0.01	−2.82, −0.38
rs28363170	−1.1	0.76	0.17	−2.60, 0.40

Cell entries are beta coefficients, standard errors (s.e.), p-values and 95% confidence intervals (CI). The HS model controlled for race/ethnicity.

In order to create a simplified score, containing the minimum number of SNPs that still explains the CES-D scores, we examined a 3-gene score with the 3 individual SNPs that had the highest individual β values (Table 4). This 3-gene score was significantly associated with CES-D score (β = −1.19, p = 0.0009). We also examined the r^2^ value for the model across the three most significant predictors and found the largest r^2^ value occurred with this 3-gene score (r^2^ = 0.092), intermediate for the 5-gene score (r^2^ = 0.085) and lowest for *DRD2/ANKK1* alone (r^2^ = 0.078), though all of these values are similar.

### Replication Results: STAR*D

Descriptive statistics are presented in Table 2. Depression scores on the HAM-D ranged from 14 to 38 (mean = 22.24, sd = 4.9). In analyses adjusted for principal components only, the genetic risk score was significantly associated with depressive symptoms (β = −0.50, p = 0.05) in the expected direction, with lower dopamine scores (indicating decreased dopamine transmission) being associated with greater levels of depression. After adjusting for all covariates, this association remained statistically significant (β = −0.51, p = 0.04; Table 3).

As noted previously, the genetic risk score in the STAR*D analyses used 3 of the 5 polymorphisms available in the analyses of the HS dataset. In order to understand the potential impact of studying only three polymorphisms, we created a score in the HS sample that used only these three polymorphisms. When using only these three polymorphisms, the genetic risk score remained significantly associated with depression in the HS dataset (β = −1.0, p = 0.004).

#### Second replication results: GSP

Depressed mood t-scores ranged from 32 to 67 (mean = 40.18, sd = 7.24; Table 2). The genetic risk score was not significantly associated with depressive symptoms in either the model adjusting only for principal components (β = −0.82, p = 0.17) or the model adjusting for all covariates (β = −0.86, p = 0.15). However, this association was in the expected direction, with higher dopamine scores (indicating increases in dopamine transmission) associated with lower levels of depression symptomatology (Table 3).

As the GSP used only 4 of the 5 polymorphisms available in the analyses of the HS dataset, we created a score in the HS sample that used only these four polymorphisms. When using only these four polymorphisms, the genetic risk score was significantly associated with depressive symptoms in the HS dataset (β = −0.79, p = 0.007).

## Discussion

Given its public health burden, there is an urgent need to better understand the etiology of depression and deploy this knowledge to inform the development and implementation of effective prevention and treatment efforts. However, depression is widely considered to be a heterogeneous disorder consisting of multiple subtypes (e.g., early vs. late onset) and symptom clusters (e.g., with vs. without vegetative symptoms), which can reflect a number of different underlying brain states [13], [73]. Noninvasive methods that capture biologically relevant inter-individual differences might be useful to better understand the complex phenotype that is depression.

Genetics offers one promising approach for identifying potential biological differences between individuals and populations. In particular, genetic investigations into the role of dopamine in depression may help identify variants that give rise to elevated susceptibility to the disorder. Prior research, from both human and animal studies, has demonstrated links between dopamine neurotransmission and depression [3], [5], [6]. Specifically, a reduction in brain dopamine has been suggested as a contributor to depressive symptoms [4], [9], [10], [11], [74], [75]. Studies have also shown that dopaminergic drugs, including pramipexole, have demonstrated efficacy in the treatment of depression [12], [76]. While these studies collectively suggest that genetic variation in the proteins related to brain dopamine neurotransmission are related to a number of behavioral traits, our study was the first, to our knowledge, to use a genetic risk score approach to examine the relationship between multiple dopamine genetic variants and depressive symptoms.

Results of the current study suggest that scores of functional polymorphisms in dopaminergic genes corresponding to reduced brain dopamine neurotransmission were significantly associated with higher levels of depression in a sample of non-depressed participants and in a large cohort of patients with depression. We also found that these genetic influences appear additive. For example, we found that the genetic risk score had a stronger association with depressive symptoms than did any individual gene. The strongest single genetic variant association was with the *DRD2/ANKK1* Taq1A Lys allele, though the p-value was weaker for this variant than that found with the genetic risk score and the 4-gene score without *DRD2/ANKK1* remained significant. Even though the individual β value was relatively large for the *DRD2/ANKK1* polymorphism, the β values cannot be compared between single genes and the multi-gene score given the differences in the range of predictor values across models (i.e., the gene score variable can range from 0–10 while the *DRD2/ANKK1* variable ranges from 0–2). The model using the 5-gene score explains more of the variance in depressive symptoms than that using *DRD2/ANKK1* alone, as evidenced by the higher r^2^ value. A strength of the genetic risk score used in the current study is that it captures several aspects of the dopamine system as a whole: levels of synaptic dopamine, modulated by polymorphisms on the genes for *COMT* and *DAT*, and binding of dopamine at three primary dopamine receptor subtypes, modulated by polymorphisms affecting these receptors. Important to the current hypotheses, the proteins encoded by the five genes we examined are abundantly present in the cortical and subcortical neural structures affected in depression [10], [11].

The score might be simplified by utilizing only the polymorphisms in *DRD2/ANKK1*, *DRD3* and *DAT*. However, the use of these 3 polymorphisms was data-driven, and therefore should be verified in independent samples. A strength of the 5-gene score is that it was an *a priori*, hypothesis-driven score based on previously published literature.

In the HS study, each one unit difference in the genetic risk score was negatively associated with a 0.80 difference in depressive symptoms. Thus, an individual with the highest possible genetic risk score (10) in this sample would be expected, based on the regression model, to have an 8 point difference in their CES-D score. This 8 point difference is clinically meaningful, as it is larger than the difference between most categories used to differentiate CES-D scores (i.e. no depression ranges from 0–9, mild depression ranges from CES-D scores of 10–15, moderate depression ranges from 16–24, and severe depression is above 25) [46].

An additional interesting aspect of the current study was that the genetic risk score was related to the severity of depressive symptoms in two groups with wide-ranging depression scores, with a lower score related to higher depression both in healthy undergraduates (HS study, mean CES-D score 8.7) and in patients with a diagnosis of depression (STAR*D, mean HAM-D score 22.2, with all participants having major depression by study design). Future studies can examine the robustness of the genetic risk score across other conditions related to dopamine neurotransmission, where it might have utility, for example, to provide insights in the setting of Parkinson’s disease, where inter-individual response to dopaminergic therapy is highly variable [77]. Theoretically, the dopamine genetic risk score could inform the likelihood that a drug with dopaminergic activity would be an effective antidepressant treatment choice for an individual patient.

Despite its strengths, the study has a number of limitations. First, there was heterogeneity across the samples, with the two replication samples having different measures of depressive symptoms and different genes comprising the genetic risk score when compared to the discovery cohort. For example, the genetic score for STAR*D was estimated based on imputed SNPs, compared to the discovery sample, which was based on genotyped SNPs. Second, replication in the second healthy adult cohort (GSP) failed to reach statistical significance, although results (Table 3) trended in the same direction as with the two other cohorts. This finding might reflect the fact that the 5-item POMS depression subscale used in the GSP, which measures only current mood state, lacked sufficient sensitivity to detect an association with the dopamine genetic risk score. It may also reflect the phenomenon known as the “winner’s curse”, whereby the effect of our genetic risk score could have been exaggerated in the discovery sample compared to the two replication studies. Future studies may be able to address this issue by examining more detailed measures of depressive symptoms or its specific features (e.g., anhedonia) as well as by examining whether the genetic risk score predicts diagnoses of depression rather than depressive symptoms. Third, the direction of correlation between the *DAT* 9/10 polymorphism and depression is in the opposite direction of that found in a meta-analysis by Lopez-Leon [30]. Mixed results are common with this polymorphism and the reason for the discrepancy is unclear. Fourth, dopamine effects are influenced by numerous factors such as the dynamics and concentration of its release [78], issues not examined in the current study. Fifth, although a great deal of evidence indicates that environmental factors may interact with genetic susceptibility to produce the final affective/behavioral phenotype, i.e, whether or not an individual will develop depression [79], [80], we did not examine gene-environment interactions in this study. Future studies might therefore assess whether dopamine genetic risk score measures interact with environmental factors in relation to depressive symptomatology, and aim to identify which environmental factors are most important. Sixth, we only examined a limited number of genetic variants related to dopamine neurotransmission. Future studies could examine whether genetic variation in proteins subserving the neurotransmission of other brain monoamines might also be important. Similarly, future efforts can examine many more sources of genetic variation related to dopamine neurotransmission, such as including additional polymorphisms in *DRD2*, *DRD4* or *DARPP-32*, for which evidence was more limited at the time the HS study was designed. Finally, the three samples differed in their makeup, calculation of the genetic risk score, and assessment of depression symptoms. Though this means that they may not reflect the truest “replication” of the original results, the varied samples more accurately reflect the heterogeneity seen in the general population and the clinical treatment of depression.

In summary, we found that a dopamine genetic risk score based on functional polymorphisms with established effects on dopamine neurotransmission was significantly associated with the level of depressive symptoms in healthy participants and with depression severity in participants with depressive disorder. This genetic risk score shows stronger associations with the measures of depression than does any single variant. Overall, the current findings support models that emphasize a role for dopamine in the pathogenesis of depressive symptoms and depressive disorder. Future research should replicate these findings and determine whether the results provide clinicians with new biological measures to improve clinical decision-making for the initiation and selection of depression therapies.
